# CMSA’s Integrated Case Management – A Manual for Case Managers by Case Managers

**DOI:** 10.5334/ijic.5534

**Published:** 2020-06-02

**Authors:** Sebastian Lindblom

**Affiliations:** 1 Division of Physiotherapy, Department of Neurobiology, Care Sciences and Society, Karolinska Institutet, Huddinge, SE; 2 Function Area Occupational Therapy and Physiotherapy, Allied Health Professionals, Karolinska University Hospital, Stockholm, SE

**Keywords:** Case management, integrated care, care coordination, continuity of care, transitional care

**Authors:** Kathleen Frasier, Rebecca Perez, Corine Latour

**ISBN:** 978-0-8261-6941-9

## Book review

Authored by Kathleen Frasier, Rebecca Perez and Corine Latour the Case Management Society of America’s Integrated Case Management manual for case managers provides a comprehensive reference manual for professional case managers across the care continuum. As stated by the authors, the book is written by case managers in favour of case managers. It provides an in-depth manual of principles and procedures for integrated case management and how it can meet the challenges of a fragmented health care system built on the medical model of treating a patient’s illness rather than each individual from a holistic viewpoint. The book presents the Case Management Society of America’s approach for case managers to fulfil the triple aim, i.e. to improve the experience and outcome of care, to improve the health of populations and to reduce costs. It delves into the roles, functions, activities and obligations of case management and is a thorough description of how case managers can assess, support and treat patients with complex needs.

The book is divided into four parts. The first part is an introduction to integrated case management and its models. The second part deals with the assessment of patient populations through integrated case management. Part three addresses social determinants and how they affect the patient’s conditions and case managers’ work, but also strategies for optimal patient communication and outcomes. The fourth and final part illustrates the barriers and facilitators for successful transitions of care together with accreditation and quality assurance of professional case management. The book includes several appendices including definitions and standards of professional case management, risk assessment and rating scales together with scoring sheets.

In part one, the first chapter takes the reader through a brief introduction to the field and the historical development of case management and goes on to explain the new U.S. 2016 Standards of Practice for Case Management. The importance of the differences in roles, functions, activities and obligations regarding case management is further described. The second chapter introduces the concept of health complexity and further describes the components and methods of the Integrated Case Management Process. Additionally, mental health parity and the importance of coordination of behavioural and mental health services are introduced. In the third chapter, the book brings a western world perspective on how the drive towards specialisation and the separation of somatic and mental health care might interfere with fulfilling the needs of patients with comorbidities and complex problems. Hence, it is emphasised that one model of case management cannot suit everyone, and therefore the needs of the individual should guide the design of the services. In order to identify and assess complex patients, two measurements are introduced. The INTERMED Complexity Assessment Grid and the INTERMED Self-Assessment Questionnaire are described and exemplified by case studies to illustrate their application.

Part two covers the assessment of different patient populations. Chapters four and five introduce the CMSA’s Integrated Case Management Complexity Assessment Grid from two perspectives in order to assess the adult and the paediatric patient. The two chapters provide a clear overview of the practice and methodology of integrated case management. Exemplified and discussed through two case studies the instruments are described in detail, covering assessment as well as possible measures to be considered appropriate within a care plan. The sixth chapter describes the practical handling of conceivable and common physical and mental conditions that a case manager may encounter. The chapter also highlights the relevance of the role of the case manager in relation to the care team and cross-disciplinary roles.

Part three of the book highlights the important aspects of achieving good population health. Chapter seven addresses social determinants of health and explains how they affect the approach and practical work of a case manager. As the authors state: “Many of the determinants are outside the scope of a case manager’s control or ability to intervene, but it is important to understand the impact” (p.142). Chapter eight handles motivational interviewing and shared decision making for the medically complex patient. A thorough review of the principles, processes and core values of motivational interviewing and shared decision making is given. In addition to theory, the chapter also contains practical tools, methods and examples that can strengthen and enrich a case manager’s work on behaviour change.

The last part of the book, beginning with chapter nine, highlights the importance of interdisciplinary teamwork and how different roles and professions should work together to develop an individualised care plan. The chapter further emphasises facilitators and barriers to successful care transitions and the central role of the case manager. The last and tenth chapter is devoted to describing the accreditation requirements and quality measures for case management and care coordination.

In summary, the book is easy to read and has an easily accessible language that invites the reader to continue reading. The book’s strengths are the practical approach with tools and methods on how case managers assess and treat patients within a complex health care system. The authors continuously weave in the case managers’ role in supporting individuals to improve their health. Furthermore, the use of case studies is a strength which facilitates understanding and translates theory into practice. The authors’ way of consistently putting patients’ perspectives at the focal point of the narrative outline the steps needed to implement and use the Integrated Case Management Approach.

The book would have benefited from a more thorough appraisal of the empirical evidence related to case management strategies in various contexts and diagnostic groups. As the book has primarily been written for the U.S. healthcare system, the potential reader should bear in mind the fact that the book has certain limitations regarding transferability to other health care contexts. That said, the book provides a broad insight into case management and how the integration of physical and mental health case management principles can be applied.

The key take-home message is that integrated care and case management, if appropriately applied, can reduce duplication, avoid gaps, improve health, enhance patient care experience and reduce costs. The book is written for case managers, who are the primary potential readers of this book, and other health care professionals that find an interest in care coordination and case management strategies.

